# Supplementary material for: Loss of SynDIG4/PRRT1 alters distribution of AMPA receptors in Rab4- and Rab11-positive endosomes and impairs basal AMPA receptor recycling
Source: Front Pharmacol. 2025 May 21;16:1568908. doi: 10.3389/fphar.2025.1568908 (PMC12133753; doi:10.3389/fphar.2025.1568908)
Supplement: Supplementary file 1 [file Supplementaryfile1.pdf]

Supplementary Material

Supplementary Figures 1-5

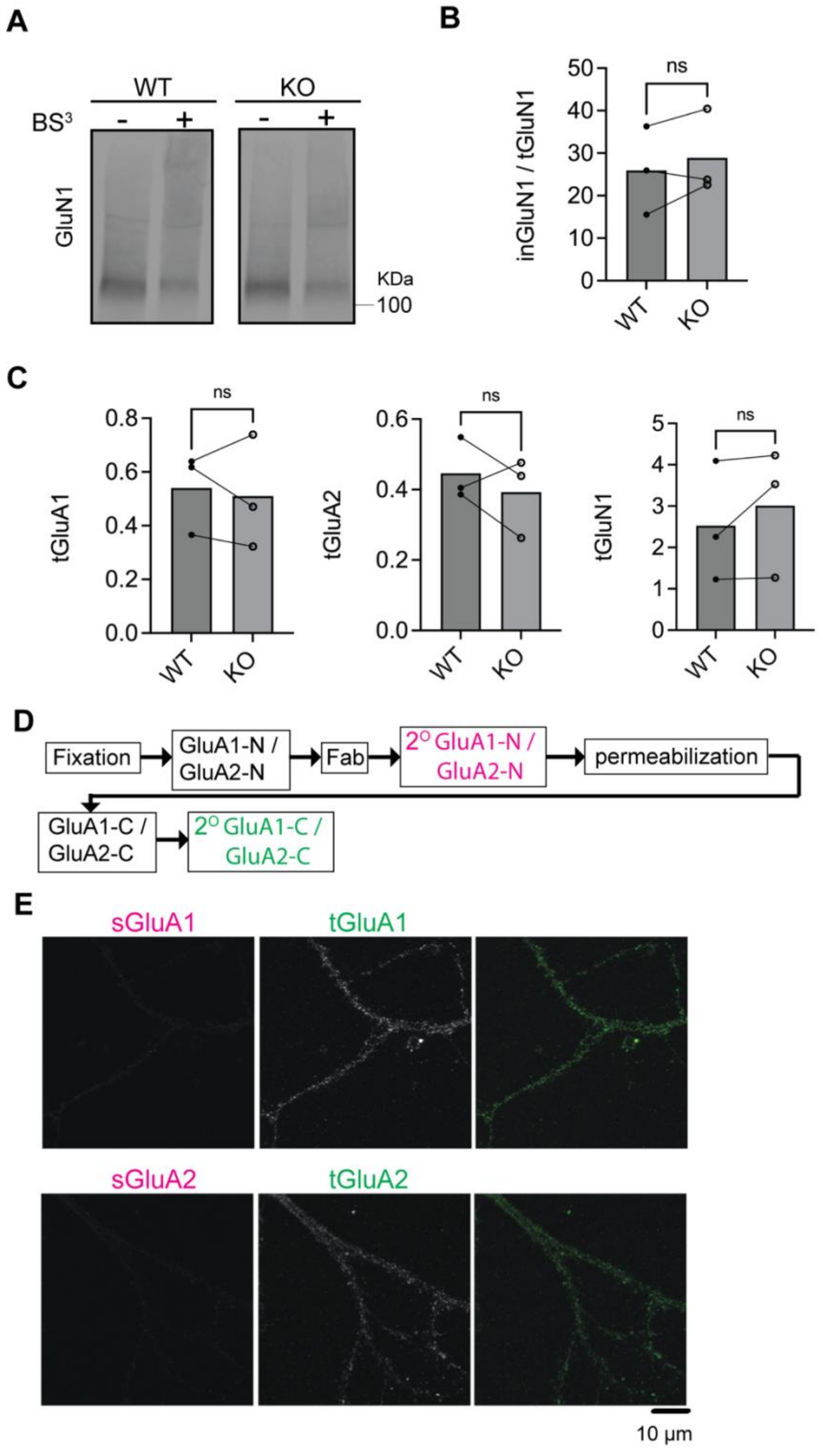

Figure S1. Fab treatment blocks binding sites for anti-mouse IgG antibodies
(A) Representative immunoblots showing intracellular GluN1 in brain slices from P14 WT and SynDIG4 KO mice after BS^3 treatment. **(B)** Graph depicts the ratio of intracellular to total GluN1 signals normalized to β-tubulin (shown in Figure 1A) in WT and SynDIG4 KO brain slices. Data are

presented as mean with statistical significance determined using ratio paired t-test; n = 3 biological replicates. **(C)** Graphs depict signals of total GluA1, GluA2 and GluN1 normalized to β-tubulin (shown in Figure 1A) in WT and SynDIG4 KO brain slices. Data are presented as mean with statistical significance determined using ratio paired t-test; n = 3 biological replicates; ns, not significant. **(D)** Schematic diagram illustrating the experimental procedure for Fab treatment. After GluA1-N or GluA2-N primary antibody incubation, Fab was applied to the neurons to block binding of fluorescently conjugated secondary anti-mouse IgG antibodies to surface GluA1 or GluA2. **(E)** Representative confocal images demonstrating that surface GluA1 (sGluA1) and surface GluA2 (sGluA2) signals are undetectable following Fab application. In contrast, total GluA1 (tGluA1) and total GluA2 (tGluA2) signals are detectable upon permeabilization and labeling with primary GluA1-C or GluA2-C antibodies and appropriate secondary antibodies. Scale bar, 10 μm.

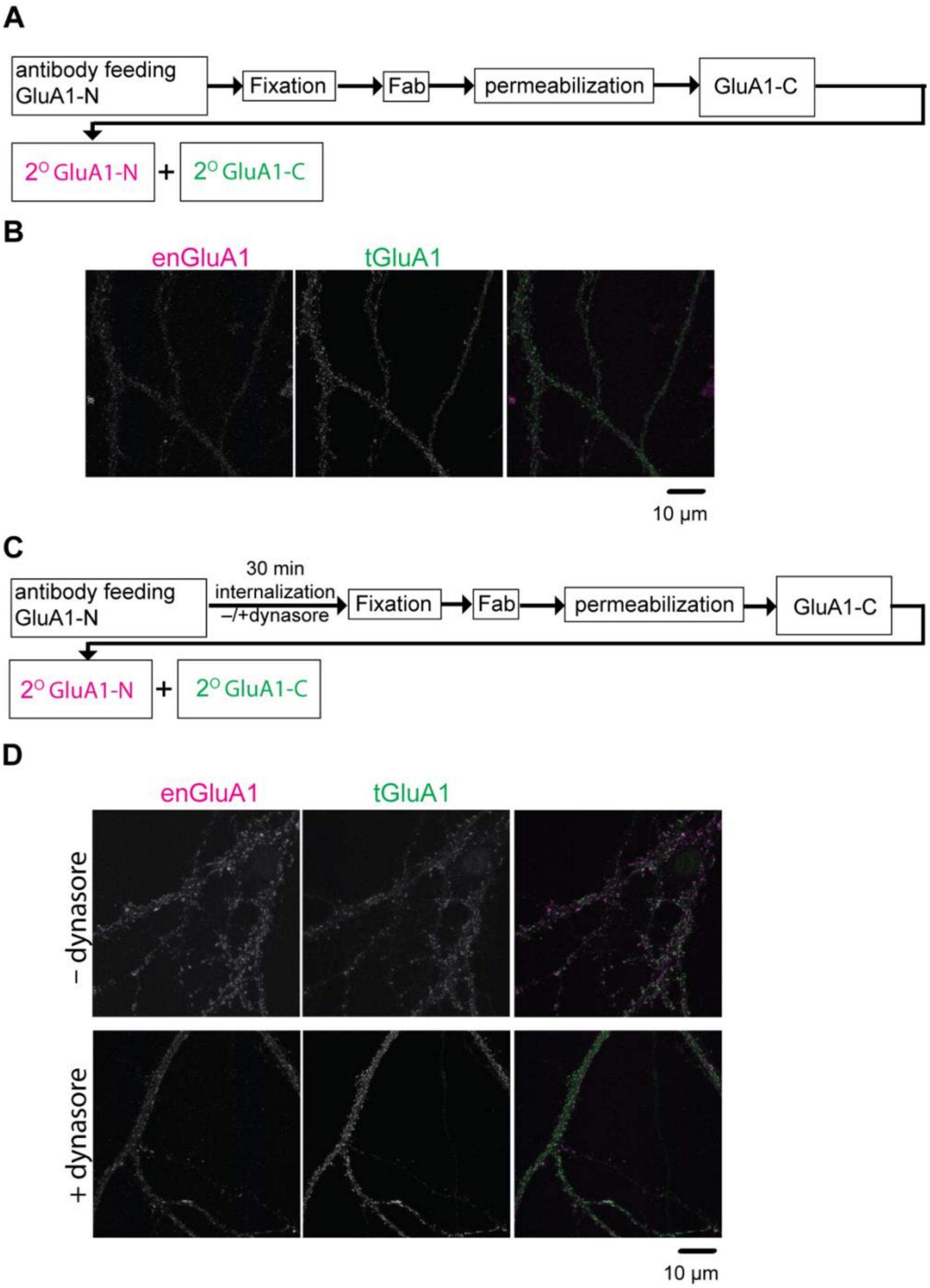

Figure S2. Dynasore inhibits dynamin-dependent endocytosis of GluA1

(A) Schematic diagram illustrating the experimental procedure. After antibody feeding (GluA1-N) at room temperature for 20 minutes, neurons were fixed and incubated with Fab antibodies to block epitopes on the cell surface. **(B)** Representative confocal images show minimal endocytosis of GluA1-N-labeled AMPARs (enGluA1) occurred during the antibody-feeding process. Total GluA1 (tGluA1) was immunostained for comparison. **(C)** Schematic diagram showing the experimental procedure where dynasore was applied during the 30 minutes internalization period. **(D)** Representative confocal images show a reduction in endocytosed GluA1 (enGluA1) signals in

neurons treated with dynasore, compared to untreated neurons, indicating that dynasore inhibits dynamin-dependent endocytosis. Total GluA1 (tGluA1) was immunostained for comparison. Scale bar, 10 µm

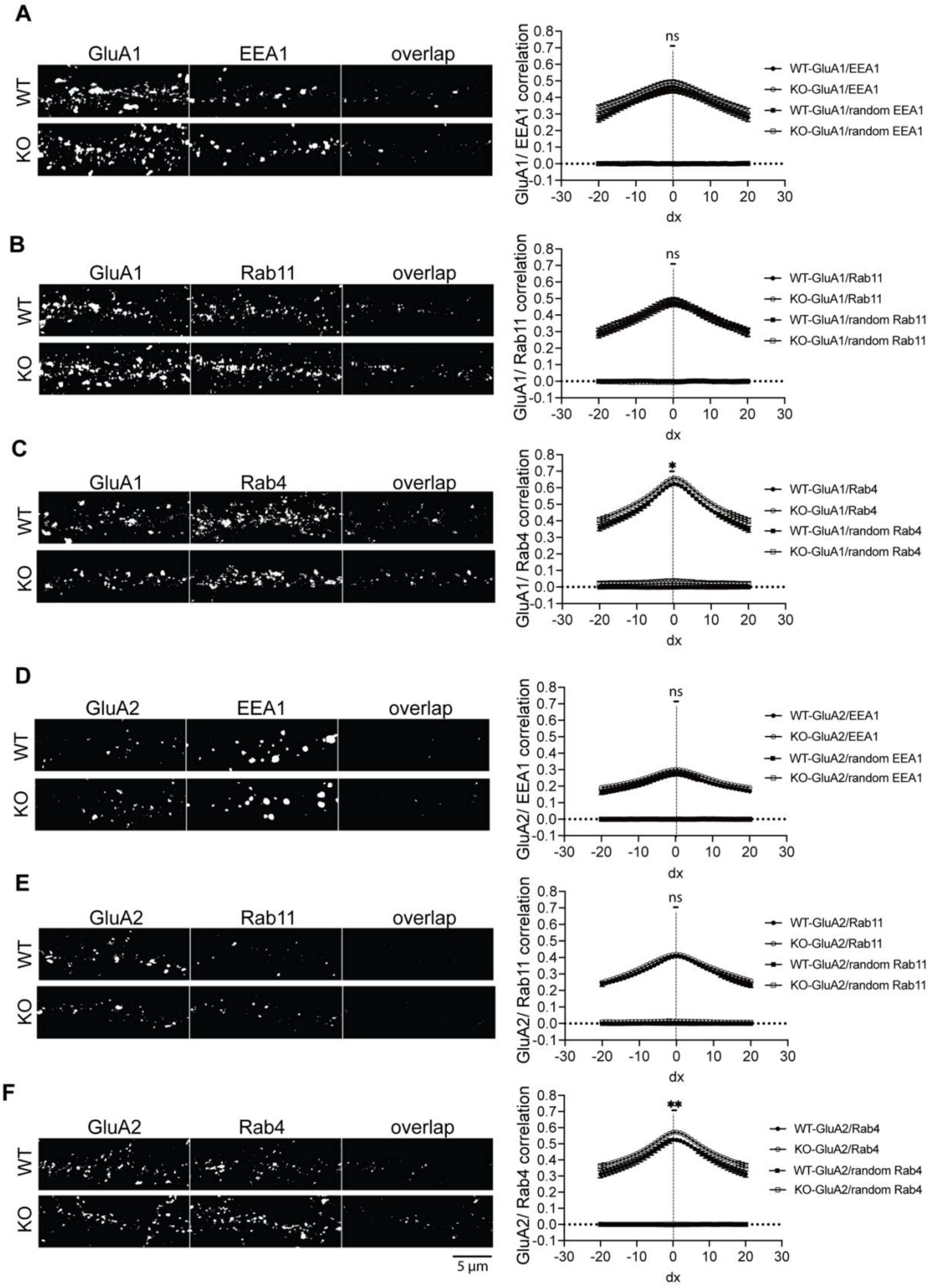
A
GluA1
EEA1
overlap
WT
KO
GluA1/ EEA1 correlation
ns
dx
WT-GluA1/EEA1
KO-GluA1/EEA1
WT-GluA1/random EEA1
KO-GluA1/random EEA1
B
GluA1
Rab11
overlap
WT
KO
GluA1/ Rab11 correlation
ns
dx
WT-GluA1/Rab11
KO-GluA1/Rab11
WT-GluA1/random Rab11
KO-GluA1/random Rab11
C
GluA1
Rab4
overlap
WT
KO
GluA1/ Rab4 correlation
*
dx
WT-GluA1/Rab4
KO-GluA1/Rab4
WT-GluA1/random Rab4
KO-GluA1/random Rab4
D
GluA2
EEA1
overlap
WT
KO
GluA2/ EEA1 correlation
ns
dx
WT-GluA2/EEA1
KO-GluA2/EEA1
WT-GluA2/random EEA1
KO-GluA2/random EEA1
E
GluA2
Rab11
overlap
WT
KO
GluA2/ Rab11 correlation
ns
dx
WT-GluA2/Rab11
KO-GluA2/Rab11
WT-GluA2/random Rab11
KO-GluA2/random Rab11
F
GluA2
Rab4
overlap
WT
KO
5 µm
GluA2/ Rab4 correlation
**
dx
WT-GluA2/Rab4
KO-GluA2/Rab4
WT-GluA2/random Rab4
KO-GluA2/random Rab4

Figure S3. Overlap between GluA1 or GluA2 and endosomal markers.
(A-C) Representative thresholded images showing the overlap of GluA1 and EEA1 (A), Rab11 (B), or Rab4 (C) in wild-type (WT) and SynDIG4 knockout (KO) neurons used for analysis in Figure 3A-C. Scale bar, 5 μm. **(D-F)** Representative thresholded images showing the overlap of GluA2 and EEA1 (D), Rab11 (E), or Rab4 (F) in WT and SynDIG4 KO neurons used for analysis in Figure 3D-F. Scale bar, 5 μm. Quantification results from Van Steensel's analysis are shown alongside the corresponding images. X-axis: dx, a horizontal pixel shift is applied to one image relative to the other. Y-axis: Pearson's correlation coefficient. Data are presented as mean ± SEM, with statistical significance determined using unpaired t-test; n = ~40 dendrite stretches for each dot shown in the graph, 2–3 dendritic stretches were cropped from individual neuron images; *$p<0.05$, **$p< 0.01$, ns, not significant.

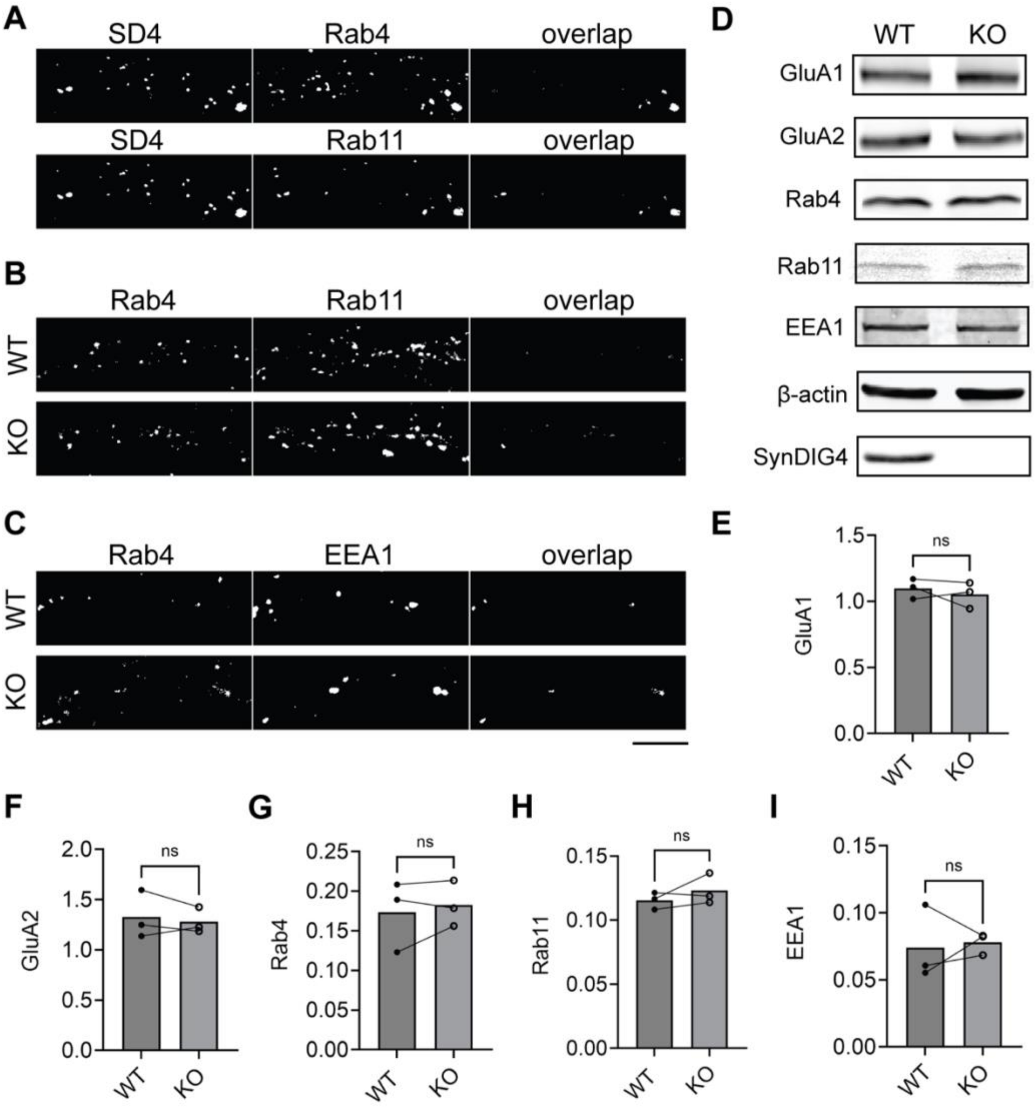

Figure S4. Overlap between SynDIG4 and endosomal markers.
(A) Representative thresholded images showing the overlap of SynDIG4 and Rab4 or Rab11 in wild-type (WT) neurons used for analysis in Figure 4A. **(B)** Representative thresholded images showing the overlap of Rab4 and Rab11 in WT and SynDIG4 knockout (KO) neurons used for analysis in Figure 4B. **(C)** Representative thresholded images showing the overlap of Rab4 and EEA1 in WT and SynDIG4 KO neurons used for analysis in Figure 4C. Scale bar, 5 µm. **(D)** Representative immunoblots showing the expression level of the indicated protein, GluA1, GluA2, Rab4, Rab11 and EEA1, in hippocampus lysates from P14 WT and SynDIG4 KO mice. **(E-I)** Quantification of GluA1 (E), GluA2 (F), Rab4 (G), Rab11 (H), and EEA1 (I) signals normalized to β-actin in WT and SynDIG4 KO hippocampal lysates. Data are presented as mean with statistical significance determined using ratio paired t-test; n = 3 biological replicates; ns, not significant.

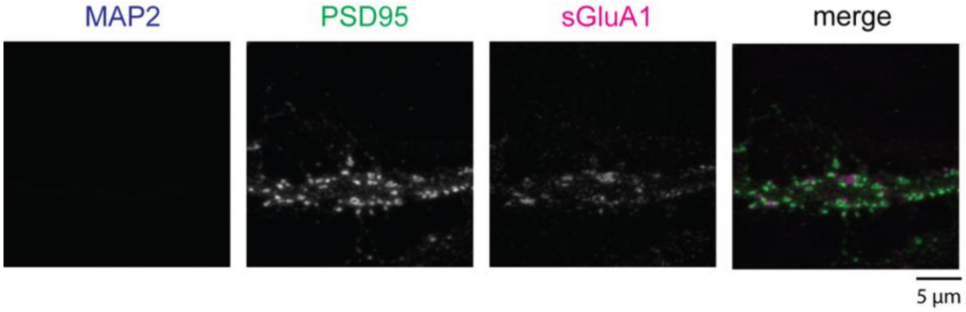

Figure S5. Surface labeling control with anti-MAP2 antibody.
Antibodies against GluA1-N and MAP2 were applied to neurons prior to permeabilization. Neurons were fixed and permeabilized followed by immunostaining with antibodies against PSD95, an intracellular synaptic scaffold, and subsequent fluorescently conjugated secondary antibodies for GluA1-N, MAP2, and PSD95. Representative confocal images demonstrate that the anti-MAP2 antibody does not penetrate the plasma membrane prior to permeabilization, confirming that the surface-labeling protocol specifically detects antigens localized to the extracellular region of the plasma membrane, while excluding intracellular epitopes. Scale bar, 5 µm.
